# Developing embedding models for cytochrome P450–ligand interaction scoring

**DOI:** 10.1093/bib/bbaf631.030

**Published:** 2025-12-12

**Authors:** Martin, Ichigaku Takigawa

**Affiliations:** Institute for Chemical Reaction Design and Discovery (WPI-ICReDD), Hokkaido University, Sapporo 001-0021, Japan; Institute for Chemical Reaction Design and Discovery (WPI-ICReDD), Hokkaido University, Sapporo 001-0021, Japan

## Abstract

Cytochrome P450 (CYP)–ligand interaction scoring plays a critical role in drug discovery and disease research by distinguishing binders from non-binders based on experimental or simulated CYP–ligand complexes. Accurate scoring of these interactions can help optimize drug dosing, efficacy, and safety. In this study, we develop robust embedding models that generate informative vector representations of CYP–ligand interactions. We focus on evaluating the transferability of these embeddings for downstream predictive tasks, particularly for the underrepresented isoforms such as CYP2B6 and CYP2C8. Using a publicly available dataset covering multiple CYP isoforms, we constructed CYP–ligand complexes via molecular docking and assigned binary activity labels based on pIC_50_ thresholds. A graph neural network (GNN) architecture was employed to learn structure-based, graph-level embeddings suitable for inhibitor classification. These embeddings were then used as input features for conventional machine learning algorithms, including tree-based ensemble methods and kernel models. Our approach eliminates the need for expert feature engineering and provides generalizable, automated descriptors of CYP–ligand interactions that can support a wide range of predictive tasks in cheminformatics.

